# Glass Film Formation on GOES Surface during High-Temperature Annealing: The Mechanism with Amorphous Phase Formation

**DOI:** 10.3390/nano12234150

**Published:** 2022-11-23

**Authors:** Mikhail L. Lobanov, Nikolai N. Nikul’chenkov, Vladimir V. Popov, Artem S. Yurovskikh, Mikhail Yu. Veksler, Vladimir I. Pastukhov

**Affiliations:** 1 Heat Treatment & Physics of Metals Department, Ural Federal University, 19 Mira St., 620062 Ekaterinburg, Russia; 2 Department of Materials and Engineering, Tel Aviv University, Ramat Aviv, Tel Aviv 6997801, Israel; 3 Institute of Nuclear Materials, Sverdlovsk Region, 624250 Zarechny, Russia

**Keywords:** GOES, glass film, forsterite, coating, X-ray diffraction, amorphous phase

## Abstract

Ceramic insulation coating (glass film) is an important constituent of grain-oriented electrical steel (GOES) designed for use in transformers. Within the scope of this study, the glass film was obtained by means of interaction between the surface of GOES containing 0.5 wt. % Cu and a heat-resistant MgO coating during annealing up to 1100 °C in the 75%H_2_ + 25%N_2_ atmosphere. The structure of glass film was analyzed using X-ray diffraction, glow-discharge optical emission spectroscopy, scanning probe microscopy, scanning electron microscopy, differential scanning calorimetry and thermodynamic calculations. After annealing, the glass film contained the following phases: crystalline (MgFe)_2_SiO_4_ and amorphous Fe-based solid solutions. The multi-stage mechanism of the glass film formation on GOES surface during high-temperature annealing was determined.

## 1. Introduction

Contemporary grain-oriented electrical steel (GOES) is a composite material that contains Fe-3%Si and has a ceramic metal film known as “the glass film” serving as an insulation coating (IC) [1,2,3,4,5]. The main purpose of IC is to create an isolation layer between sheets of magnetic cores. Owing to IC, each core coil loop serves as an individual magnetic flow conductor, thus minimizing eddy-current losses due to higher electrical resistance of the material at decreased thickness of core sheets [1,3]. In addition, IC protects the metal surface against corrosion. Moreover, it creates elastic tension stresses in GOES crystallites, which decrease the magnetic losses due to the size reduction of the domain structure [3,6].

The formation of IC on GOES is a multi-stage process that includes several thermochemical treatment operations [2,7]. GOES production consists of the following four main stages, which have a significant impact on the IC formation [2,7,8,9]: decarburizing annealing (DA), heat-resistant coating, high-temperature annealing (HTA) and the application of an electrical insulation coating.

As a prerequisite for obtaining a high-grade IC, the glass film layer that forms on the steel surface during HTA should have the required thickness and a certain chemical composition [1,3]. The glass film formation depends on many factors determined at the previous treatment stages. The quality of the resulting coating after HTA is determined by thickness, structure and chemical composition of the oxidized layer. These parameters, in turn, are determined by the conditions of decarburization annealing: the temperature, time and composition of the atmosphere (an oxidation potential). The study [6] demonstrated that both an increase in the decarburization annealing temperature in the range of 740–950 °C and an increase in pH_2_O/pH_2_ in the range of 0.28–0.58 increase the amount of subsurface fayalite, which affects the future magnetic properties of the material after the forsterite coating formation.

Glass film formation under HTA is also a multi-stage process that includes several solid-phase reactions occurring in a wide range of temperatures [1,3,7,8,9]. The formation of GOES surface structure begins as oxidation during DA [3,7,8,9,10,11,12,13,14,15,16]. An ultrathin layer, mainly consisting of silicon oxide SiO_2_ coated with (FeMn)SiO_3_/(FeMn)_2_SiO_4_ shells, forms on the steel surface after DA [12,13]. Behind it there is a Si-depleted region of the α-Fe solid solution. A certain decrease in the oxygen content is also observed in this region. At a depth of ~0.5 μm from the surface, SiO_2_ oxides have a near-spherical shape. In addition, this region is characterized by the maximum concentrations of Si and O. Behind this area there is a zone of 1–2 µm in length, where the oxides are in the form of large plates, and small particles are also observed. This area transforms rather abruptly into a matrix solid solution.

Thermodynamic calculations were performed in order to draw Fe–Si, FeO–SiO_2_ and MnO–SiO_2_ phase diagrams [17]. The surface layer of GOES has been determined to be in the (α + γ) double-phase region at the DA temperature. According to both calculated and experimental data [17,18,19,20,21], the main phases in the oxide surface layer are FeO, FeSiO_3_, Fe_2_SiO_4_, MnSiO_3_, Mn_2_SiO_4_, SiO_2_ and combinations thereof.

After DA, an aqueous suspension of MgO is applied to the steel surface, which is then dried at temperatures ≤500 °C. The powder remaining after the evaporation of free H_2_O (the heat-resistant coating) contains chemically-bound moisture that usually constitutes 3–5 wt. % [3,6,7,14]. In order to prevent steel sheets from sticking together during HTA, it is required that partially hydrated magnesium oxide is present on the GOES sheet surface: MgO in the Mg(OH)_2_ shell, to which a certain number of water molecules are attached by hydrogen bonds—Mg(OH)_2_·nH_2_O.

HTA is carried out in bell-type furnaces in order to obtain a coarse-crystalline (110) [001] texture and refine the metal and create a surface glass film that mainly consists of Mg_2_SiO_4_ [3,8,20]. Metal coils with an MgO coating are annealed at a heating rate of 10–25°/h first up to ~650 °C in an atmosphere of hydronitric mixture, then up to ~1150 °C in a dry hydrogen atmosphere. The heating rate ranges and pH_2_O/pH_2_ in the furnace atmosphere are important HTA parameters that affect the final phase composition of the surface and, therefore, have an impact on the glass film structure. The quality of GOES electrical insulation coating applied during the next stage is determined by the homogeneity of the chemical and phase compositions of the glass film after HTA.

The glass film is formed as a result of the interaction between MgO particles and the steel surface oxidized after DA: the layer formed during HTA is a brittle glass-like film. The studies [3,7,14,20] showed that the final IC was double-layer system, whereby the forsterite (Mg_2_SiO_4_) layer had been formed first and then coated with aluminum orthophosphate. The thickness of IC is ~4 µm according to [7].

The studies [22,23] demonstrated that a thermally stable amorphous phase is formed in the glass film surface after a full cycle of HTA in the temperature range of α → γ transformation. Based on the thermodynamic calculations, an assumption has been made about chemical interactions leading to the formation of an amorphous phase and its estimated chemical composition has been suggested.

Despite the fact that the GOES production technology was developed a significant amount of time ago, the processes occurring on the alloy surface remain largely unclear, at least in detail. This study aims to determine the mechanism underlying the formation of glass film on the GOES surface during HTA.

A clearer understanding of the formation mechanism of glass film is required in order to determine the best possible ways to improve its quality. Such findings can provide means to control the surface layer structure, preventing the onset of the factors that cause defect formation and impair the electrical insulation properties of the coating and the magnetic properties of GOES.

In this study, we investigate the process of glass film formation on the GOES surface at various stages of HTA with the effect caused by amorphous phase formation taken into account.

## 2. Materials and Methods

The study was conducted on production alloy samples of 0.70 mm thick cold-rolled strip containing the following: 0.03 wt. % C, 3.1 wt. %, 0.54 wt. % Cu, 0.3 wt. % Mn, 0.014wt. % Al, 0.01 wt. % N, 0.05 wt. % Cr, 0.05 wt. % Ni, 0.006 wt. % S, 0.01 wt. % P, and 0.003 wt. % Ti (the remainder comprising Fe and unavoidable impurities). The chemical composition corresponded to the Russian GOES (Fe-3%Si-0.5%Cu) production specifications [24,25].

The samples were subjected to DA for 8 min at 840 °C in a 75%H_2_ + 25%N_2_ atmosphere humidified to the dew-point temperature of +69 °C [7]. After the annealing, the carbon content did not exceed 0.002 wt. %. Subsequently, the samples were rolled down to the thickness of 0.25 mm.

The samples were coated with aqueous MgO suspension prepared using MgO Scora powder (Israel) with particle size of 0.5–10 µm and volume fraction of particles below 1 µm in size constituting ~25%. The suspension was prepared by means of intense mixing of MgO and H_2_O using a mixer at 5–10 °C. The application of the suspension was carried out by dipping with subsequent rubber roll aided excess removal. The coating was air-dried at 250 °C for 5 min.

The dried suspension of MgO + Mg(OH)_2_·nH_2_O was subjected to differential scanning calorimetry (DSC) using Netzsch STA 449 C Jupiter instrument at heating rate of 30°/min.

The coated samples were subjected to annealing in separate areas of a laboratory tube-type gradient furnace up to 600, 700, 800, 900, 1000 and 1100 °C in a 75%H_2_ + 25%N_2_ atmosphere with dew-point of −40 °C at a constant heating rate of 15°/h from 20 up to 1100 °C with subsequent cooling in the furnace. The obtained samples were used in the subsequent investigation.

X-ray structure analysis (XRD) of the coated sample surface after annealing was performed on a Bruker D8 Advance diffractometer using Kα Co radiation. The XRD measurements were each carried out with a temperature increment of 50 °C during heating from 30 up to 900 °C, and then with an increment of 25 °C when heating from 900 up to 1050 °C; then during cooling from 1050 down to 850 °C, the temperature step was 25°, and it was 50 °C when cooling from 850 down to 30 °C. Electron diffraction phase analysis was performed using the Bruker DIFFRAC.EVA VB program, searching for matched interplanar spacings in the ICDD PDF-2 database (2019).

An integral chemical analysis of the material layers from the surface into the depths of the samples was carried out using a glow discharge optical emission spectroscopy (GDOES) analysis by GDA-750 glow discharge analyzer [18,19]. The surface of the sample after the GDOES was almost ideally etched [18,19], which made it possible to use the GDOES analyzer as a tool for surface preparation with etching to a very shallow depth.

In order to theoretically estimate the phase composition of metal surface layers at high temperatures, thermodynamic equilibria were calculated using ThermoCalc software.

SMM-2000 scanning probe microscope (PROTON Plant, Moscow, Russia) was used to carry out structural studies at different levels from the sample surface (~0, 1, and 5 μm); cantilevers (probes) from Brucker (USA) with a tip radius of 2 nm were used.

Sample layers at various depths from the surface were etched to ~1 and 5 μm depth using GDA-750. The obtained surfaces were also investigated by means of scanning electron microscopy (SEM).

SEM structure studies were performed using a Tescan Mira3 SEM at an accelerating voltage of 20 kV. The EBSD HKL Inca with Oxford Instruments analytical system was used for local phase identification. The phase composition analysis was carried out by comparing the Kikuchi diffraction patterns with the reference patterns using Oxford Instruments. The analysis was conducted point-by-point, with the entire diffraction patterns recorded with an increased duration of exposure.

## 3. Results and Discussion

In order to clarify the structural changes observed in the MgO + Mg(OH)_2_·nH_2_O coating and to explain the effect of the structure on the processes occurring on the Fe-3%Si alloy surface, DSC was used for the heat-resistant coating powder removed from the metal surface.

**Figure 1:**
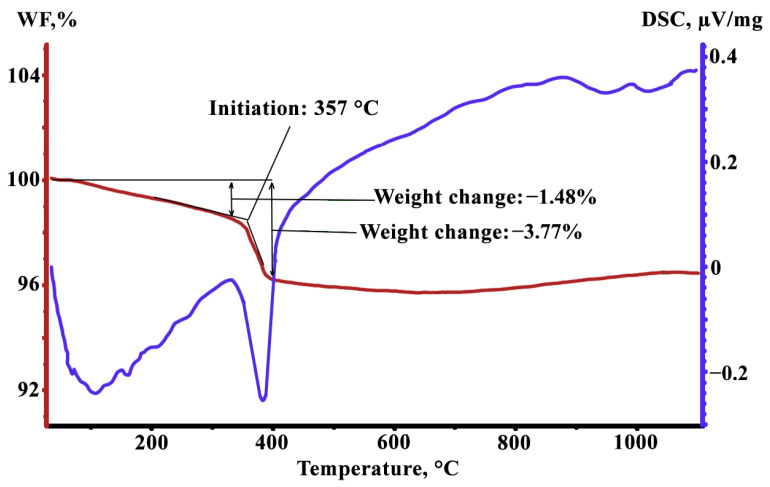
DSC of MgO + Mg(OH)_2_·nH_2_O at a heating rate of 20°/min, WF—Weight Fraction.

The calorimetric measurements show (Figure 1, blue curve) that a slight smooth heat release occurs at 25–360 °C; a dip in the curve corresponding to the absorption of a relatively large amount of energy is observed at 360–400 °C; then the release of heat occurs again at a slower pace and the process damps completely at ~850 °C. The comparison of the calorimetric data with the results of the sample weight control (Figure 1, red curve) suggests that molecular bounded H_2_O evaporates at heating up to ~360 °C; and at 360–400 °C, decomposition of Mg(OH)_2_ takes place with the release of MgO and H_2_O, and then water also evaporates.

Thus, at temperatures of 360 to 400 °C, a large amount of H_2_O vapor is released on the metal surface in the heat-resistant powder coating. The released H_2_O oxidizes the GOES surface to form FeO. In the surface of the material, all silicon was oxidized to SiO_2_ as early as at the DA stage [11]. According to [14], a certain amount of FeO is necessary to form Fe_2_SiO_4_ with further transformation to Mg_2_SiO_4_, which is the main component of the glass film. It was emphasized [7,22] that FeO can be mutually dissolvable with MgO with the formation of (FeMg)O, which is probably the only mechanism by which Mg can penetrate deep into the material.

**Figure 2:**
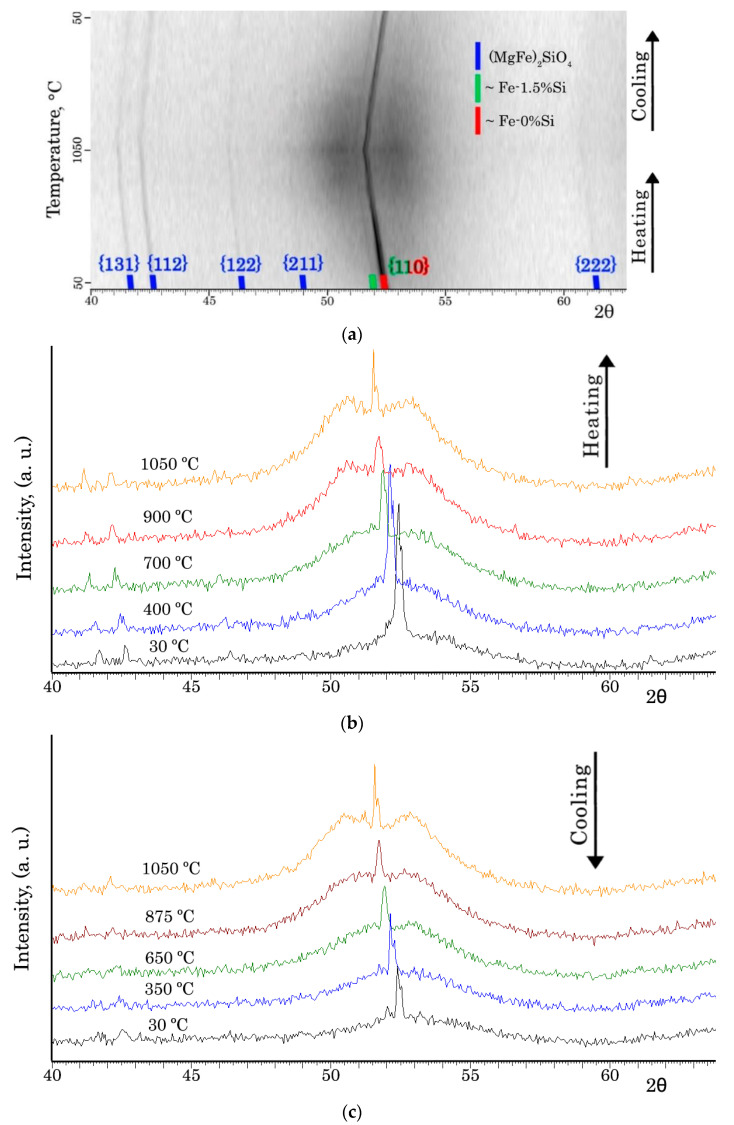
KαCo diffraction pattern of coated Fe-3%Si-0.5%Cu alloy after HTA: (**a**) during the entire processing cycle; (**b**,**c**) during heating and cooling, respectively.

XRD was used with heating within the temperature range of 30–1050 °C (Figure 2) to determine the phase composition of the IC surface after complete HTA. Two Fe-based solid solutions with different silicon contents (~0 wt. % and ~1.5 wt. %) and forsterite (MgFe)_2_SiO_4_ were recorded (Figure 2a). In addition, a halo was detected, indicating the presence of an amorphous phase in the surface. At the same time, a certain amount of the amorphous phase was retained at cooling (Figure 2c). Moreover, at high temperatures the halo was divided into two (Figure 2b,c). It presumably corresponds to two amorphous phases with slightly different chemical compositions. These results are consistent with the experimental data reported in [22,23].

**Figure 3:**
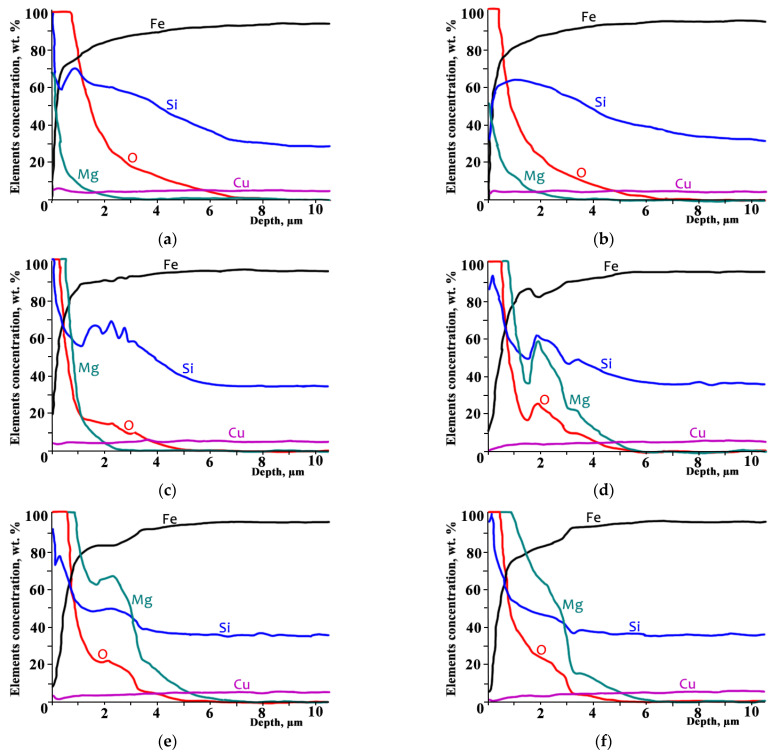
Distribution of elements in the surface layer of Fe-3%Si-0.5%Cu alloy with MgO coating after annealing at T ≤ N °C, where N = (**a**) 600 °C; (**b**) 700 °C; (**c**) 800 °C; (**d**) 900 °C (**e**) 1000 °C; (**f**) 1100 °C; the scale: Fe is ×1, Si, Mg, O, Cu is ×0.1.

According to the GDOES analysis (Figure 3) intense interaction occurred between GOES sample surface and heat-resistant coating during heating.

The initial non-monotonic distribution curve of silicon (Figure 3a) was predominantly formed during DA at temperatures above 800 °C [7,11,18,19]. During the DA process, oxygen was diffusing into steel, which was accompanied with a slower diffusion of Si towards the surface and a constant formation of SiO_2_ и Fe_2_SiO_4_. The total amount of Si contained in the surface during HTA remained almost unchanged (Figure 3). The amount of surface Si decreased and the flattening of its distribution curve was observed at ~700 °C (Figure 3b). At 800 and 900 °C (Figure 3c,d), the Si distribution curve became significantly non-monotonic and then flattened again at higher temperatures (Figure 3e,f).

The total amount of oxygen in GOES surface decreased during heating from 600 up to 800 °C (Figure 3a–c), it increased at 900 °C (Figure 3d) and remained almost unchanged at higher temperatures. The oxygen distribution curve demonstrated non-monotonic behavior at 900 °C (Figure 3d) and then flattened somewhat at higher temperatures (Figure 3e,f).

The total magnesium amount in the surface increased within the investigated range of 600–1100 °C; the increase was more significant when temperature exceeded 800 °C (Figure 3c). Mg penetrated the surface layer to a depth of ~5 µm, which practically coincided with the oxidation zone thickness after DA. The Mg distribution curve behavior became non-monotonic abruptly at 900 °C and flattened at higher temperatures (Figure 3e,f).

The copper distribution curve changed insignificantly and remained practically flat throughout the whole annealing temperature range; its content decreased in proximity to the surface due to the increase in the quantity of other elements (Figure 3). Manganese distribution behavior in the GOES surface (not demonstrated in Figure 3) was similar to that of Cu within the entire annealing temperature range. Mn concentration within 5 µm from the surface was low and did not exceed 0.2 wt. %, which was comparable to the GDOES analysis sensitivity level. Its concentration increased up to ~0.3 wt. % rapidly in the deeper layers of GOES.

It is important to note that within 1 µm from the surface, iron was the main component (over 70 wt. %) at all annealing temperatures (Figure 3). Therefore, it can be argued that the internal oxidation zone obtained by means of DA is predominantly a solid solution within the depth that exceeds 1 µm from the sample surface.

We find it significant that during the annealing of coated GOES, additional oxidation of the surface initially took place presumably in the form of FeO and oxygen diffusion into GOES due to the presence of H_2_O that had been released during Mg(OH)_2_ decomposition. Further annealing in the atmosphere containing a large amount of hydrogen led to a partial reduction in oxides, transfer of elements from oxides into solid solution and their diffusion along the present concentration gradients accompanied with gradual flattening of their distribution curves. Therefore, the non-monotonic distribution curves of Si, O, Mg, Fe at some annealing temperatures and their subsequent flattening occurred due to element bounding into oxides (their exclusion from the diffusion process) and oxide decomposition (inclusion of the elements into the diffusion process). Cu remained in the solid solutions due to its low level of affinity for oxygen. Presumably, Mn also remained in solid solutions and did not significantly participate in the oxidation processes.

The ratios of the elements in their concentration distributions over the depth of the analyzed layers suggested that, in the low-temperature range, wustite FeO had been predominantly formed in the surface (Figure 3a). Later, MgO was dissolved in it with the formation of magnesiowustite (FeMg)O. At temperatures above 900 °C, a phase in the form of an almost continuous layer was observed in the surface (~0.2 μm), which, according to the ratio of elements, could be identified as forsterite (MgFe)_2_SiO_4_ (Figure 3e,f).

Findings regarding the chemical composition of each layer allowed calculation of the possible thermodynamic equilibrium phase composition and comparison thereof with the phase composition and structure observed in the layer.

The GDOES data were used to simulate the thermodynamic equilibrium phase compositions of the surface layers of Fe-3%Si at various HTA temperatures. To this end, the distributions of elements at each temperature (Figure 3) were selected at certain distances from the surface and were transferred to ThermoCalc software (in wt. %) to calculate the phase equilibria of a given chemical composition at the various temperatures.

**Figure 4:**
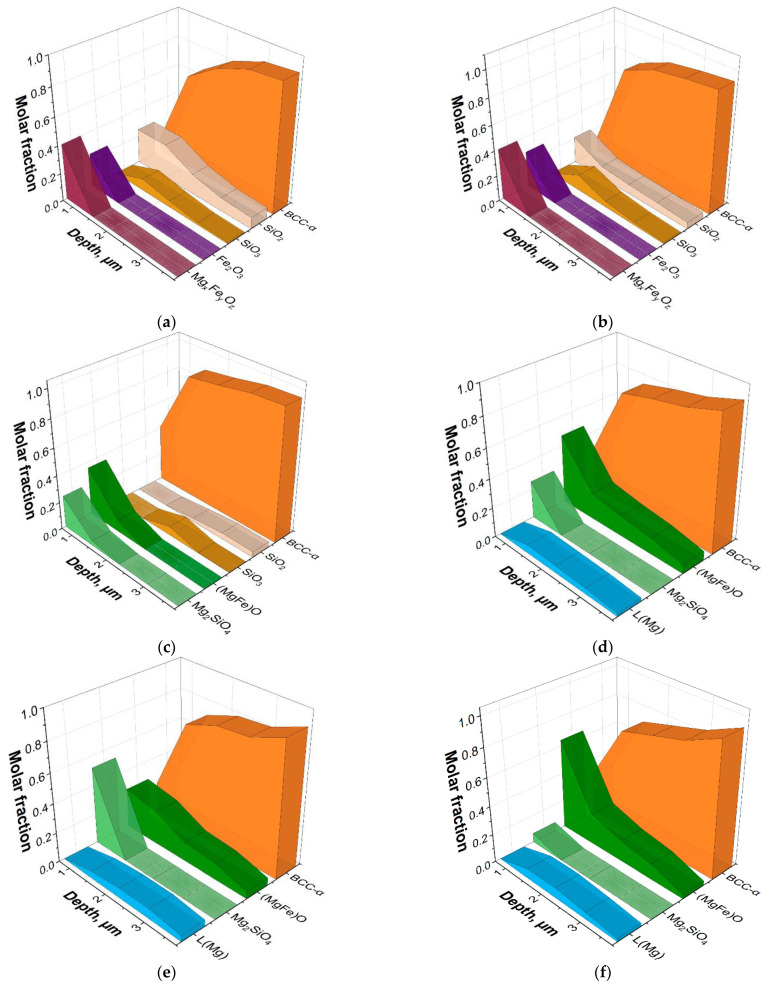
Results of thermodynamic calculations of possible phases in the surface layer of the Fe-3%Si-0.5%Cu alloy at temperatures: (**a**) 600 °C; (**b**) 700 °C; (**c**) 800 °C; (**d**) 900 °C; (**e**) 1000 °C; (**f**) 1100 °C.

The calculation of phase equilibria showed that at 600, 700 °C, in the surface (up to a depth of 1.5 μm), there were oxide phases of Mg_x_Fe_y_O_z_ type (close to magnesiowustite (FeMg)O), Fe_2_O_3_, SiO_2_, as well as metastable oxide SiO_3_, the maximum amount of which was observed at a depth of 1.5 µm. In addition, at these temperatures, an increase in the amount of Fe(Si) solid solution is observed from ~0.1 molar fraction at a depth of 0.7 μm to ~0.8 molar fraction at a depth of 1.5 μm. In the context of this study, it is important to emphasize the pronounced oxidation of the Fe-3%Si surface with the appearance of the (MgFe)O type oxide film. Obviously, this oxidation occurs due to H_2_O evaporating from the heat-resistant coating and massively appearing in the process of Mg(OH)_2_ decomposition. Apparently, the oxidation of the surface layers to form FeO oxide allows the MgO molecules to dissolve in it with the formation of magnesiowustite (Figure 4a,b). This assumption is consistent with the results reported in [7].

At 800 °C, Mg_2_SiO_4_, MgO, and Fe-based solid solutions are in the phase equilibrium in the surface layer. In the deeper layers, silicon oxides are retained (Figure 4c). There are no Fe-based oxides in the surface layer, presumably due to the absence of an oxidizing agent (H_2_O) in the environment. This assumption is also confirmed by the fact that the integral amount of oxygen in the surface layer of the metal keeps decreasing as the temperature increases up to 700 °C and then changes insignificantly (Figure 3).

At a temperature of 900 °C, Mg_2_SiO_4_, MgO and the solid solution are in equilibrium in the surface layer. At a depth of about 1.5 μm, the ThermoCalc calculations show the presence of almost pure magnesium (~0.1) in the liquid state. Thus, all oxygen in the surface region of the material turns out to be bound into stable oxides; at the same time, however, there appears to be not enough oxygen to oxidize all the magnesium present in the system. Magnesium does not dissolve in the Fe-based solid solution and, therefore, is released as a pure element being in the liquid state at the given temperatures (T_m_ = 650 °C) (Figure 4d). Obviously, such a large amount of pure Mg should have been detected by XRD, which was not the case. The reason for this may be the dissolution of Mg in the metastable solid solution of iron with the formation of amorphous phase.

A further increase in the calculation temperatures up to 1000, 1100 °C leads to a decrease in forsterite content and an increase in MgO content in the subsurface layers and, at the same time, to the presence of magnesium in a liquid state in a layer 1 μm deep from the surface.

**Figure 5:**
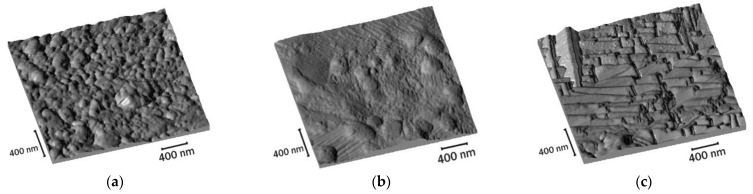

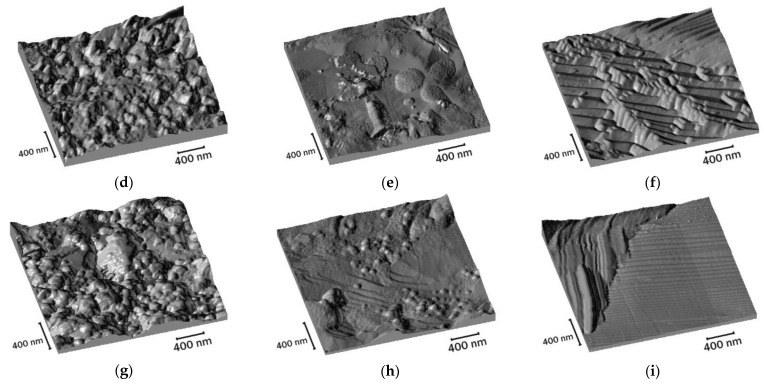
Microstructure of Fe-3%Si-0.5%Cu layer surfaces with MgO coating after annealing at different temperatures: (**a**–**c**) 700 °C; (**d**–**f**) 800 °C; (**g**–**i**) 1000 °C; (**a**,**d**,**g**) the surface; (**b**,**e**,**h**) the surface depth about of 1 µm; (**c**,**f**,**i**) the surface depth about of 5 µm.

Scanning probe microscopy was carried out to study the surface microstructure of the heat-resistant coating at different depths. The “structural” reliefs were shown to be significantly different depending on both the surface layer depths as well as the annealing temperature (Figure 5). At a depth of ~5 μm, a geometrically-structured relief, corresponding to the crystallographic structure of the solid solution, was recorded.

At ~1 µm depth, rounded precipitates are present in the matrix; its relief partially coincides with the grain surface. With an increase in temperature up to ~900 °C, larger individual precipitates were observed and smaller precipitates appear, as compared to the initial precipitates observed at low temperatures. Moreover, on the surface of larger particles, its own microrelief was observed. At a temperature of ~1000 °C, the majority of precipitates are the smaller ones.

The samples at 700 °C show a rather uniform surface relief (~0 μm), which is characterized by a grain size of ~20 nm. An increase in temperature to 800 and 1000 °C leads to a noticeable difference in the grain size with a corresponding increase in the surface roughness (Figure 3a,d,f).

The results of GDOES and the ThermoCalc calculations (Figure 3 and Figure 4) allow us to state that the phase composition of the material changes from layer to layer with an increasing temperature: new phases are formed, while some phases disappear; the compositions of the phases change, and their proportions are different depending on the depth. Presumably, the oxides Fe_2_O_3_, Mg_x_Fe_y_O_z_, SiO_2_ and the metastable oxide SiO_3_ are converting into the oxides (MgFe)O and Mg_2_SiO_4_. At temperatures above 900 °C, ThermoCalc calculations show the presence of almost pure magnesium which should be in a molten state. Evidently, in this case, the thermodynamic calculations of the ideal phase equilibria do not predict a possibility of a metastable amorphous phase formation in the solid state.

According to an assumption made in [22], the most dispersed oxide phases (MgFe)_2_SiO_4_, (MgFe)O dissolve within the temperature range of metastability of the α-Fe crystal lattice (α → γ phase transformation). At the same time, Mg atoms transfer to the solid solution, thus forming strong chemical bonds with Si atoms. The regular structure of the crystal lattice of the alloy disappears as a large number of similar complexes are randomly located in the solution. This state was observed to be quite stable both during heating and cooling because of the large number of strong chemical bonds between the elements.

**Figure 6:**
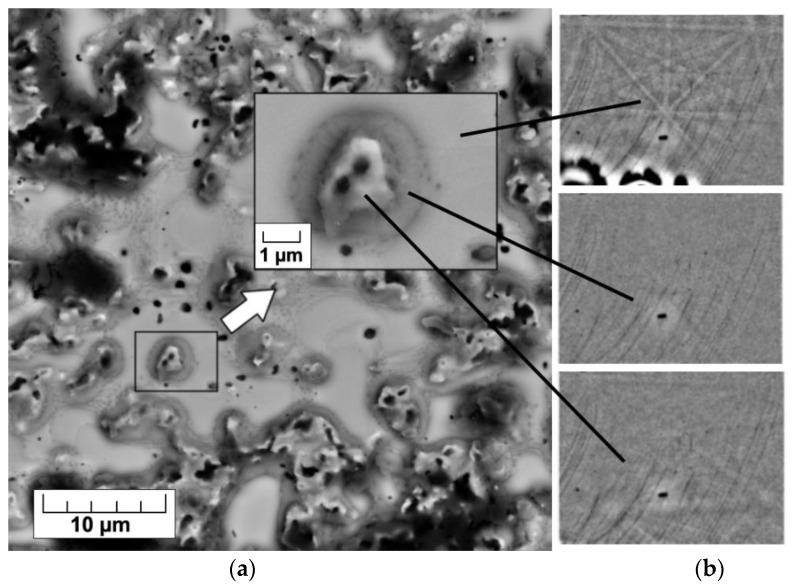
SEM image of the surface (~1 µm deep from the surface) of Fe-3%Si-0.5%Cu alloy with MgO coating after HTA (**a**) and diffraction Kikuchi-like pattern from different sites (**b**) (the upper pattern is the BCC-lattice diffraction, the lower pattern is the forsterite lattice).

Orientation microscopy based on electron backscatter diffraction did not identify the amorphous phase detected earlier by the XRD. To determine the areas of amorphous phase formation, local phase identification was possible from different sites on the same surface of Fe-3% Si alloy with a heat-resistant coating after HTA (Figure 6). SiO_2_ particles (the darkest) are covered with a forsterite shell. The amorphous phase can be assumed as an intermediate layer between these oxide complexes and the solid solution matrix, since this region has no clear diffraction Kikuchi-like pattern (Figure 6b, the middle insert).

**Figure 7:**
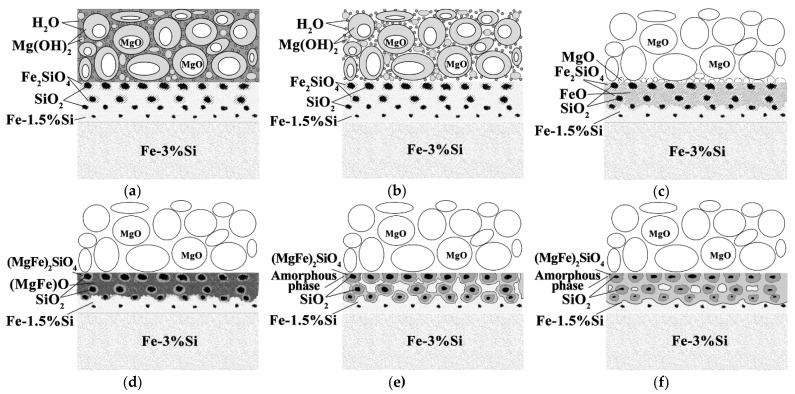
Possible glass film formation scheme on the Fe-3%Si-0.5%Cu alloy surface during HTA: (**a**) after applying the MgO suspension at about 10 °C; (**b**) after drying the MgO suspension at about 150 °C; (**c**) heating at 300–700 °C; (**d**) heating at 700–900 °C; (**e**) heating at 900–1150 °C; (**f**) holding at 1150 °C.

Considering both the results of this study and the published data [7], it can be assumed that the glass film layer formation during HTA can be divided into the following overlapping stages (Figure 7):

1. Evaporation of free H_2_O occurs (Figure 7a,b) when drying a suspension of the heat-resistant coating;
2. The H_2_O molecules, weakly bound to the main components of the heat-resistant coating, are removed from the heat-resistant coating at about 25–360 °C. Mg(OH)_2_ is decomposed with a massive release of H_2_O at 360 °C. Due to the release of H_2_O from the coating, the surface of the solid solution is oxidized to form FeO and Fe_2_O_3_ iron oxides. FeO becomes the predominant oxide with the increase in the amount of oxygen in the surface (Figure 7c);
3. At 700–900 °C, MgO molecules are dissolved in FeO to form (MgFe)O (Figure 7d). Diffusion interaction of (MgFe)O with the Fe_2_SiO_4_ shells of SiO_2_ particles leads to the formation of (MgFe)_2_SiO_4_;
4. Further heating above 900 °C occurs in a strongly reducing pure dry hydrogen atmosphere. The amount of oxygen in the system drops dramatically. In the coating layers adjacent to the solid solution, relatively low-oxygen (MgFe)_2_SiO_4_ oxides are partially retained, and almost pure Mg is formed, according to the thermodynamic calculations. Since pure magnesium was not observed experimentally, it can be assumed that Mg reduced from the oxide phases dissolves in a metastable (due to the proximity of α → γ-transformation temperature) iron-based solid solution with the formation of the amorphous phase (Figure 7e);
5. It is suggested that the amount of amorphous Fe-based solid solution increases during holding in the dry hydrogen atmosphere (Figure 7f).

The findings of this study are sufficiently consistent with the study [3], that includes a suggested glass film formation mechanism for the temperature range of 1000–1200 °C and demonstrates surface microstructure after HTA at 800–1200 °C. The main focus of the study [3] is the forsterite formation and its dominating role as a phase in GOES surface.

## 4. Conclusions

In this study, we investigated the process of glass film formation on the GOES surface during various stages of high-temperature annealing. After HTA, the glass film on the surface of GOES (Fe-3%Si-0.5%Cu) contained the following phases: crystalline and amorphous Fe-based solid solutions, as well as (MgFe)_2_SiO_4_. It was demonstrated that MgO transferred to the GOES surface by dissolving in FeO. The iron oxide was formed by means of surface oxidation by water vapor, which had been released during the decomposition of Mg(OH)_2_.

The mechanism of the glass film formation on the surface of GOES during HTA was suggested. This mechanism includes a series of thermochemical reactions, which occur on the GOES surface during HTA: (I) at 400–700 °C, the iron in the surface is oxidized to FeO; (II) at 700–900 °C, MgO dissolves in FeO to form magnesiowustite (MgFe)O; (III) at 700–900 °C, (MgFe)O interacts with SiO_2_ to form (MgFe)_2_SiO_4_; (IV) at 900–1150 °C, the thermostable amorphous phase is formed in the surface layer. The amorphous phase grows with temperature increase, while the amounts of (MgFe)_2_SiO_4_ and SiO_2_ decrease. Some amount of this phase remains under cooling down to room temperature. 

The findings regarding the glass film formation mechanism allows for the control of the GOES surface structure, eliminating the onset of factors that decrease the levels of the electrical insulation properties of the coating and the magnetic properties of GOES. The suggested mechanism accompanied with the specification of all alterable HTA parameters (temperature, time, atmosphere oxidation and reduction potential, amount of water in heat-resistant coating) should facilitate obtaining defect-free glass film of required thickness on GOES.

The observed amorphous solid solutions of a Fe-Si-Mg system in the surface layers of GOES having undergone HTA are also of scientific and practical interest. Presumably, these solid solutions are formed due to solid-state amorphization as a result of thermodynamic equilibrium processes and are retained during cooling down to room temperature.

## Data Availability

Not applicable.
